# Model for the Controlled Synthesis of O-Antigen Repeat Units Involving the WaaL Ligase

**DOI:** 10.1128/mSphere.00074-15

**Published:** 2015-12-30

**Authors:** Yaoqin Hong, Peter R. Reeves

**Affiliations:** School of Molecular Bioscience (D17), The University of Sydney, New South Wales, Australia; Swiss Federal Institute of Technology Lausanne

## Abstract

The Wzx/Wzy O-antigen pathway involves synthesis of a repeat unit (O unit) consisting of 3 to 8 sugars on an inner-membrane-embedded lipid carrier. These O units are translocated across the membrane to its periplasmic face by Wzx, while retaining linkage to the carrier, and then polymerized by Wzy to O-antigen polymer, which WaaL ligase transfers to a lipopolysaccharide precursor to complete lipopolysaccharide synthesis, concomitantly releasing the lipid carrier. This lipid carrier is also used for peptidoglycan assembly, and sequestration is known to be toxic. Thus, O-unit synthesis must involve precise regulation to meet demand but avoid overproduction. Here we show that loss of WaaL reverses a known growth defect in a *Salmonella* mutant that otherwise accumulates O-unit intermediates and propose that WaaL is also involved in a novel feedback mechanism to regulate O-unit synthesis, based on the availability of O units on the periplasmic face of the membrane.

## OPINION/HYPOTHESIS

**FIG 1:**
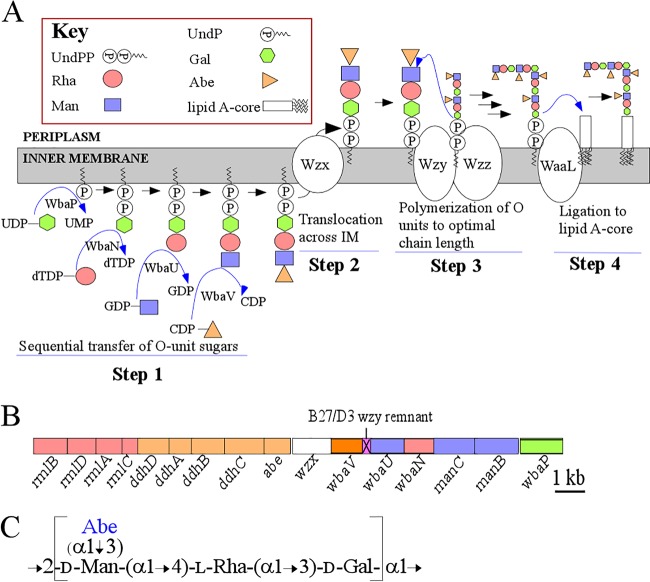
*S. enterica* group B1 O-antigen synthesis. (A) Biosynthetic pathway. Step 1, transfer by WbaP of galactose-1-phosphate (Gal-P) to Und-P to give UndPP-linked Gal, followed by other glycosyltransferases that build the rest of the O unit sequentially; step 2, Wzx translocation of the O unit across the membrane; step 3, polymerization at the reducing end of a growing UndPP-linked O-unit polymer by Wzy, in effect extending the chain length by one for each cycle; step 4, the ligation reaction catalyzed by WaaL incorporating O antigen into LPS. (B) Genetic map of the O-antigen gene cluster. Pathway genes and products are color coded for each nucleotide sugar precursor. (C) Group B1 O-unit structure (2). Note that the side branch moiety, Abe, shown in blue is missing in Δ*abe* O units.

Production of long-chain O antigen is essential for the survival of many Gram-negative bacteria and has major implications for their ability to colonize and establish infection. The Wzx/Wzy pathway, which is the most common for O-antigen synthesis (1), begins with addition of the first sugar as a sugar phosphate to undecaprenyl phosphate (UndP) by an initial transferase (IT), to give UndPP-sugar in the inner membrane, with the sugar on the cytoplasmic face. In the *Salmonella enterica* group B1 serovar Typhimurium strain LT2 used in this work, the IT is WbaP and the sugar is galactose (Gal) (2). Other sugars are added by glycosyltransferases to generate complete O units of 3 to 8 sugars (1), which are translocated to the periplasmic surface by Wzx, where they can be polymerized by Wzy to give a polymer (Fig. 1A). Both the monomer and polymer can be ligated to lipid A-core by WaaL (3) to generate complete lipopolysaccharide (LPS) for export to the outer membrane. The O unit is also used, after modification, for assembly of O-antigen capsule, made in large amounts under some conditions (4). See Fig. 1 for O-unit structure and synthesis.

Mutants that produce incomplete O units can show growth defects (5). We have shown in a collection of mutants, including an *S. enterica* Δ*abe* strain, that produce incomplete forms of the O units that this lethality is associated with Wzx being inefficient in the translocation of the unnatural substrate (6–8). The situation for *wzx* mutants is complicated by the fact that they can be difficult to obtain (9–11). We found that an *S. enterica* Δ*wzx* strain has a growth problem and that cells lyse if O-antigen synthesis is allowed (8); in the case of *Pseudomonas aeruginosa*, cells may accumulate second-site mutations, which either restore the synthesis of normal LPS or abolish O-antigen expression completely (12). These findings give strong indications that *wzx* mutants are deleterious if the O unit is being made, but we are not aware of controlled experiments that establish this.

However, no deleterious effects have been reported for Δ*wzy* mutants of the same species, and none were observed in other Δ*wzy* constructs that we have made (13). It was reported that a Δ*wzy* mutant has a 20-fold reduction in the overall number of O units in the LPS compared to the number in its wild-type counterpart (14), and a 10-fold difference was later reported by our group (15). Thus, a *wzx* or an *abe* mutant that cannot translocate the O units at all or does so with severely retarded efficiency, using no O units or almost none, is very deleterious, whereas a *wzy* mutant which uses 5 to 10% of the normal amount of O units (14, 15) has no ill effects at all. It seems that O-unit synthesis is deleterious only if translocation is either absent or inefficient and that otherwise cells adjust synthesis to match demand. There were also several *Salmonella* Δ*waaL* strains constructed previously (6, 8, 16), showing that *waaL* is not essential, although no growth details were reported. It was also shown that in the absence of a suitable acceptor for ligation, the UndPP–O-unit concentration was maintained at about the same level as observed in the wild type (17).

**FIG 2:**
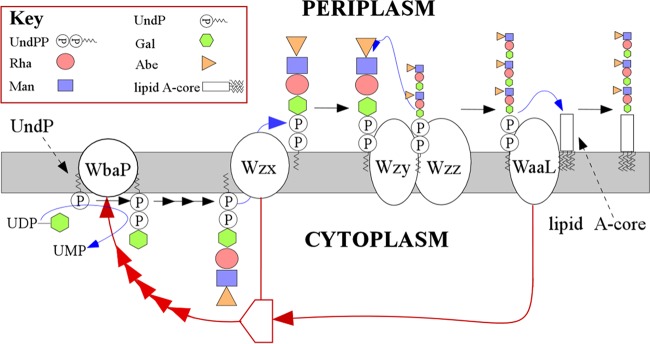
Working model for the regulation of O-unit synthesis. We propose that WaaL sends a feedback signal to WbaP, based on its level of contact with UndPP-linked material in the periplasmic face of the membrane to facilitate the synthesis of more O units. Note that some LPS molecules have only single O units or non-modal-length O-antigen chains, and these are not shown in the figure. Wzx is shown as being involved in bringing the WaaL and WbaP proteins in contact; however, more evidence is required to understand the nature of the proposed molecular machinery.

Here, we show for *S. enterica* LT2 that the accumulation of either O units or a trisaccharide intermediate (Fig. 1C) is deleterious only if accumulation is on the cytoplasmic face of the membrane. We also demonstrate that a *waaL* deletion can reverse the deleterious effects of accumulating O-unit intermediates. We propose that O-unit synthesis is regulated to maintain a consistent level on the periplasmic face of the inner membrane and propose a model in which this is achieved by WaaL regulation of WbaP activity (Fig. 2).

### Assessment of O-antigen mutant growth in a Gal-free M9-based medium.

Assessment of growth of *S. enterica* O-antigen mutants requires a Gal-free medium, with the major carbon source not leading to catabolic repression (e.g., glucose). This allows controllable O-antigen synthesis by the addition of Gal (see the next section for details). Here we used 1× M9 minimal medium supplemented with 100 µM CaCl_2_, 1.6 mM MgSO_4_, 0.32% (wt/vol) Casamino Acids, 0.4% (vol/vol) glycerol, 1 µg ⋅ liter^−1^ thiamine, and 8 µg ⋅ liter^−1^ tryptophan. Overnight cultures were diluted 1:40 and grown at 37°C with aeration. Tetracycline was added after the first 30 min to induce expression of genes carried by either the pWQ552 or the pWQ572 vector (7). To allow for O-unit expression, Gal (0.2%, wt/vol) was added after another 30 min. Samples were taken every 30 min for 6 h for measurement of optical density at 600 nm (OD_600_) with a BioPhotometer (Eppendorf). The growth of each strain under each tested condition was repeated three times (error bar shows standard errors of the means [SEM]). Samples were then transferred to test tubes and photographed to illustrate turbidity.

### Accumulation of O-antigen repeat units affects growth only when they are on the cytoplasmic face of the membrane.

We constructed our strains in a Δ*galE* background (8), as described by Datsenko and Wanner (18) (see Table S3 in the supplemental material for the oligonucleotides used). This prevents the endogenous synthesis of UDP-Gal, the precursor substrate used by WbaP to give UndPP-Gal. This approach allows experimental control of O-antigen synthesis by addition of exogenous Gal (8). The *abe*, *wzx*, *wzy*, and *waaL* genes were deleted by allelic replacements (Fig. 1B). The sequences of the resulting constructs were confirmed by showing that complementation with a clone of the deleted gene restored the wild-type phenotype in all cases (Fig. S1 and S2).

10.1128/mSphere.00074-15.1 Figure S1  SDS-PAGE profiling of LPS from *S. enterica* group B LT2 and derived strains. Bacterial strains were diluted 1:20 from overnight cultures in modified M9 medium that uses glycerol and Casamino Acids as the carbon sources and is free of galactose (see the text) and were grown at 37°C. Tetracycline is added after 30 min for expression of genes carried by either pWQ552 or pWQ572, as described previously (7), and Gal was added to a final concentration of 0.2% (wt/vol) at an OD_600_ of ~0.3 to allow O-antigen synthesis. Cells were harvested after another 60 min of growth. LPS was prepared and analyzed as described previously (8). Download Figure S1, TIF file, 1.9 MB. Copyright © 2015 Hong and Reeves. 2015 Hong and Reeves This content is distributed under the terms of the Creative Commons Attribution 4.0 International license.

10.1128/mSphere.00074-15.2 Figure S2  Adding a plasmid that harbors a respective gene deletion can reverse growth defects in either the Δ*abe* or the Δ*wzx* mutant. Thus, the growth defect observed for the two strains are specifically due to the two genes deleted. Strains were grown in a modified M9 medium that uses glycerol and Casamino Acids as the carbon source and that is free of galactose (see the text). Galactose is added after 1 h to allow for O-antigen synthesis. A growth assay was performed as described in the text. Error bars represents SEM derived from three repeats of the experiment. Download Figure S2, PDF file, 0.1 MB. Copyright © 2015 Hong and Reeves. 2015 Hong and Reeves This content is distributed under the terms of the Creative Commons Attribution 4.0 International license.

**FIG 3:**
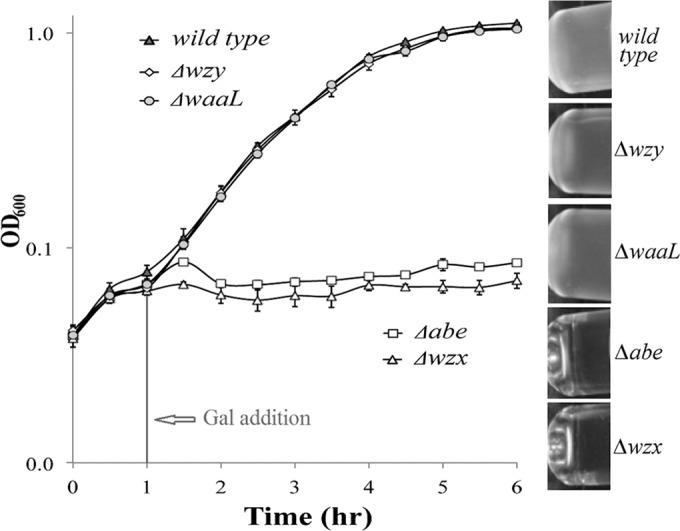
Disruption of O-antigen synthesis can be either conditionally lethal or nonlethal. Growth assays were performed as described in Materials and Methods (averages are from 3 experiments, and error bars show SEM). (Left) Growth curve; (right) turbidity.

All of the mutants grew well in the absence of Gal (Fig. S3). However, the Δ*abe* and Δ*wzx* mutants stopped growing after exposure to Gal for 30 min, whereas both the Δ*wzy* and Δ*waaL* mutants were unaffected (Fig. 3). The normal growth in the absence of Gal shows that the growth effects are directly related to O-unit synthesis. Both Wzy and WaaL act after translocation (1), and mutants will accumulate any surplus O units on the periplasmic face of the membrane, whereas the Δ*wzx* mutant will accumulate them on the cytoplasmic face (9), and it has been shown that the trisaccharide intermediates produced in Δ*abe* mutants are mostly not translocated (6, 8). The growth defect is correlated with an accumulation of O units or intermediates on the cytoplasmic face of the membrane. We therefore propose that there is a feedback mechanism that regulates the synthesis of O units, based on the level of O units on the periplasmic face of the inner membrane.

10.1128/mSphere.00074-15.3 Figure S3  Disruption of O-antigen genes shows lethality only if O-unit synthesis is turned on. Strains were grown in a modified M9 medium that uses glycerol and Casamino Acids as the carbon source and that is free of galactose (see the text). A growth assay was performed as described in the text. Error bars represent SEM derived from three repeats of the experiment. Download Figure S3, PDF file, 0.1 MB. Copyright © 2015 Hong and Reeves. 2015 Hong and Reeves This content is distributed under the terms of the Creative Commons Attribution 4.0 International license.

### WaaL may have a role in the regulation of O-unit synthesis.

The proposed feedback mechanism requires detection of O units on the periplasmic face of the inner membrane and transmission of a signal. This most likely involves regulation of the functioning of O-unit synthesis machinery. Regulation commonly acts on the first step in the synthesis of the compound being regulated, which in this case is the synthesis of UndPP-Gal by WbaP. It is unlikely that regulation is by interaction of the end product, the UndPP-O unit, with the promoter, as synthesis occurs on the cytoplasmic face of the inner membrane and the end product is proposed to be effective in regulation only when it is on the periplasmic face of that membrane.

WaaL, which ligates single O units or polymers to lipid A-core, the last step in LPS synthesis, is a potential candidate for involvement in this regulation. We note that the UndPP-O unit is also a substrate for O-antigen capsule synthesis but that control is likely to be based on O-antigen demand, as long-chain O antigen is present under all conditions, whereas the presence of O-antigen capsule is variable (4). We hypothesize that WaaL regulates O-unit synthesis by transmitting a signal determined by the availability of UndPP-linked single or polymeric O units, which controls the function of WbaP.

**FIG 4:**
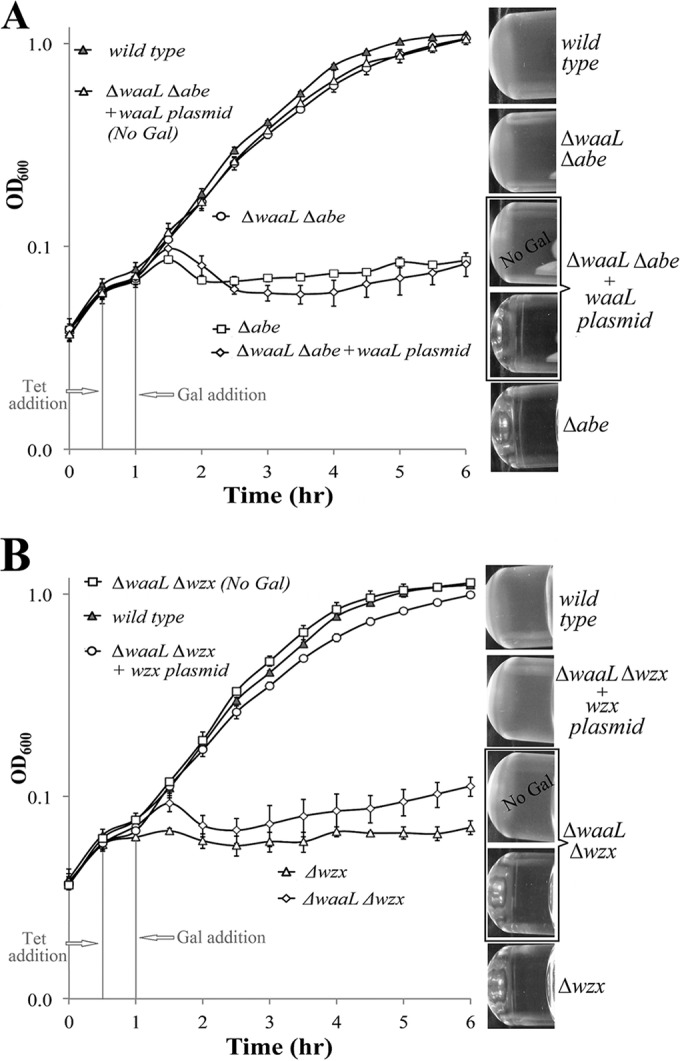
WaaL is required for the growth defect observed in a Δ*abe* strain but not in a Δ*wzx* strain. Growth assays were performed as described for Fig. 3. (A) WaaL is dependent for growth on the defect of *abe* deletion*. *(B) Deletion of *waaL* could not reverse the growth defect of *wzx* deletion. (Left) Growth curve; (right) turbidity.

We constructed a Δ*waaL* Δ*abe* double mutant by allelic replacement of the *abe* gene in the Δ*waaL* strain (see Table S1 in the supplemental material for strains and plasmids). The SDS-PAGE profile (Fig. S1, lane 11) was the same as for the Δ*waaL* strain (Fig. S1, lane 9), as expected. Strikingly, the strain grew normally even in the presence of Gal, which allows initiation of O-unit synthesis (Fig. 4A). To exclude the possibility that this observation relates to suppressor mutations inactivating O-unit synthesis, we introduced a *waaL* clone that regenerates a typical Δ*abe* strain in terms of its LPS SDS-PAGE profile (Fig. S1, lane 12) and growth defect pattern (Fig. 4A). We conclude that WaaL activity is required for the *abe* deletion to be deleterious.

10.1128/mSphere.00074-15.4 Table S1  Strain and plasmid table. Download Table S1, PDF file, 0.1 MB. Copyright © 2015 Hong and Reeves. 2015 Hong and Reeves This content is distributed under the terms of the Creative Commons Attribution 4.0 International license.

10.1128/mSphere.00074-15.5 Table S2  Oligonucleotides for cloning. Download Table S2, PDF file, 0.04 MB. Copyright © 2015 Hong and Reeves. 2015 Hong and Reeves This content is distributed under the terms of the Creative Commons Attribution 4.0 International license.

10.1128/mSphere.00074-15.6 Table S3  Oligonucleotides used for strain constructions. Download Table S3, PDF file, 0.1 MB. Copyright © 2015 Hong and Reeves. 2015 Hong and Reeves This content is distributed under the terms of the Creative Commons Attribution 4.0 International license.

It is commonly proposed that O-antigen synthesis membrane proteins, including Wzx, Wzy, and Wzz, form a complex (19–22), and recently Wzy and Wzz were isolated as a complex in a pulldown experiment (23). The probable presence of such a complex has important implications for our proposed model. WbaP, which initiates O-unit synthesis, is also a membrane protein and may be part of the proposed protein complex (Fig. 2) and affect the control of O-unit synthesis. We note that there are data suggesting that one class of mutant WbaP proteins retains association with the developing O-unit until its completion (24), which raises the possibility that control of O-unit synthesis may operate by WbaP not releasing completed UndPP-linked O units for translocation.

However, the situation is more complex, as in a Δ*waaL* Δ*wzx* strain, the absence of WaaL does not relieve the growth defect of the Δ*wzx* mutant, which is still unable to grow when O-antigen synthesis is initiated (Fig. 4B). It may be that Wzx is required for transmission of a regulatory signal from WaaL to WbaP. It is too early to speculate on details, as the nature of any complex is unknown, but it is clearly possible that if one component (e.g., Wzx) is missing from a complex, then another component (e.g., WaaL) might lose contact with the target protein, perhaps WbaP.

### Conclusions.

We propose that maintaining the balance between supply and demand of O units for O-antigen synthesis requires a specific regulatory process and that this involves a sufficiency of O units being detected by the periplasm-exposed part of the WaaL ligase. This is a novel hypothesis based on limited data, but those data are all consistent with the proposed model at this stage. We studied only *S. enterica*, but the findings from *P. aeruginosa* mutants referred to above, namely, that they accumulate second-site mutations which either restore synthesis of normal LPS or abolish O-antigen expression completely (12), suggest that similar regulatory processes occur in other species. The ability to adjust O-unit supply based on its levels on the periplasmic side of the membrane allows O-antigen chain length to be varied depending on circumstances and may thus have very important implications, for example in the tradeoff between phage resistance and virulence (25)

Our growth curve data give a clear demonstration of the effect of mutation in *wzx* on growth and also the effect of blocking completion of the O unit. The finding that a *waaL* mutation allows a strain lacking a functional *abe* gene to grow well when making an incomplete O unit shows that WaaL has some novel activity in addition to its ligase action and is consistent with the proposed model. However, the recognition that WaaL may play an important role is but the first step in understanding this physiological process, which most likely involves a protein complex with multiple functions.

### Possible directions.

We have proposed a novel model for the regulated synthesis of O units based on interactions within a hypothetical membrane protein complex. Many fundamental aspects of this model will need to be rigorously tested and validated. In Table 1, we provide some sample questions yet to be addressed.

**TABLE 1:** Possible directions for future investigation of the proposed model

Question	Reasoning
Is O-unit synthesis regulated?	The proposal that O-unit synthesis is well regulated is based on only a few observations. More-extensive genetic and biochemical studies are needed.
Does WaaL regulate O-unit synthesis?	Additional data are needed to show that WaaL is directly involved in the regulation of O-unit synthesis.
How is regulation achieved?	The model suggests that the regulation proposed most likely involves interactions between multiple proteins, but evidence is lacking. The possibility that regulation may involve transcriptional/translational adjustments will need to be investigated.
Does the proposed protein complex really exist?	We proposed that regulation is achieved by interactions in a multiprotein complex. However, only Wzy and Wzz have been confirmed to interact (23).
How do UndPP-linked O-unit monomers and polymers interact with WaaL ligase?	Can we isolate WaaL in association with its substrates, and can we crystallize WaaL and ultimately view it in its various modes of actions?
