# Supplementary figures and images for: Model for the Controlled Synthesis of O-Antigen Repeat Units Involving the WaaL Ligase
Source: mSphere. 2015 Dec 30;1(1):e00074-15. doi: 10.1128/mSphere.00074-15 (PMC4863624; doi:10.1128/mSphere.00074-15)

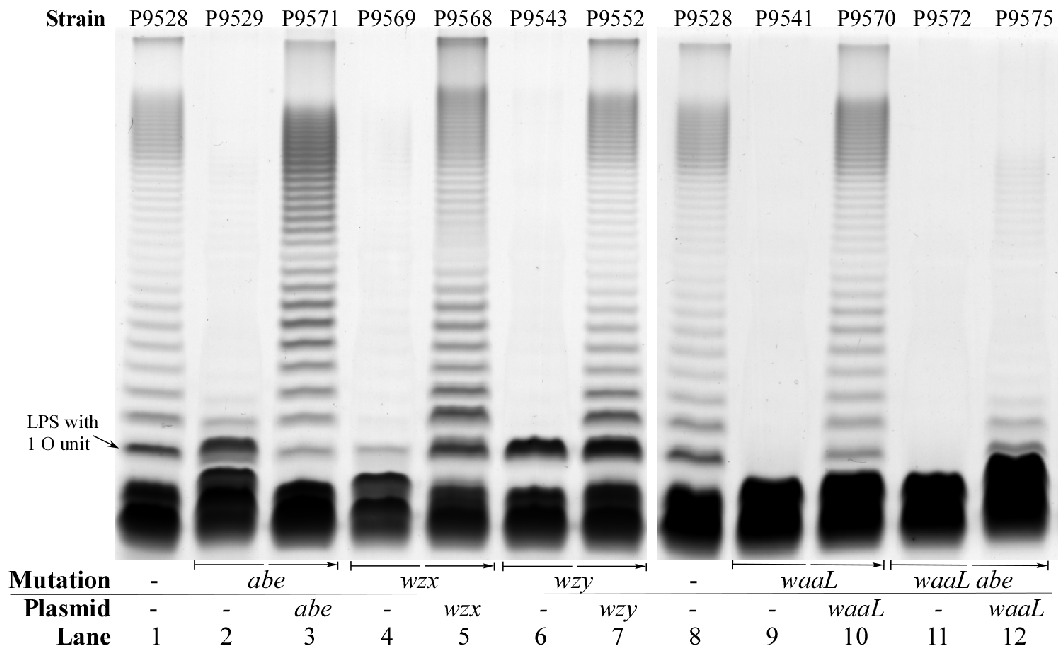

Supplement: Figure S1 [file sph001160055sf1.tif]

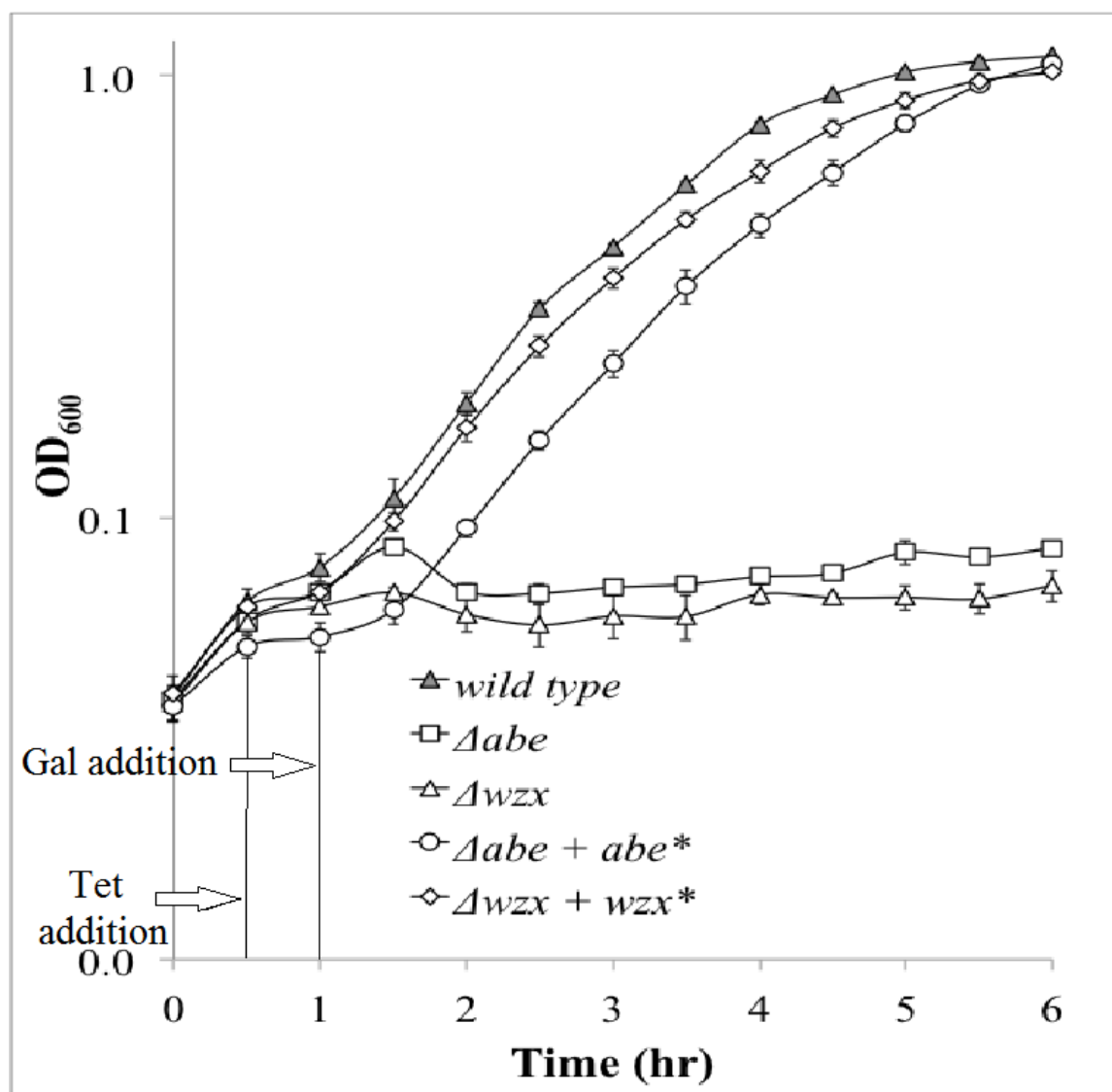

Supplement: Figure S2 [file sph001160055sf2.pdf]

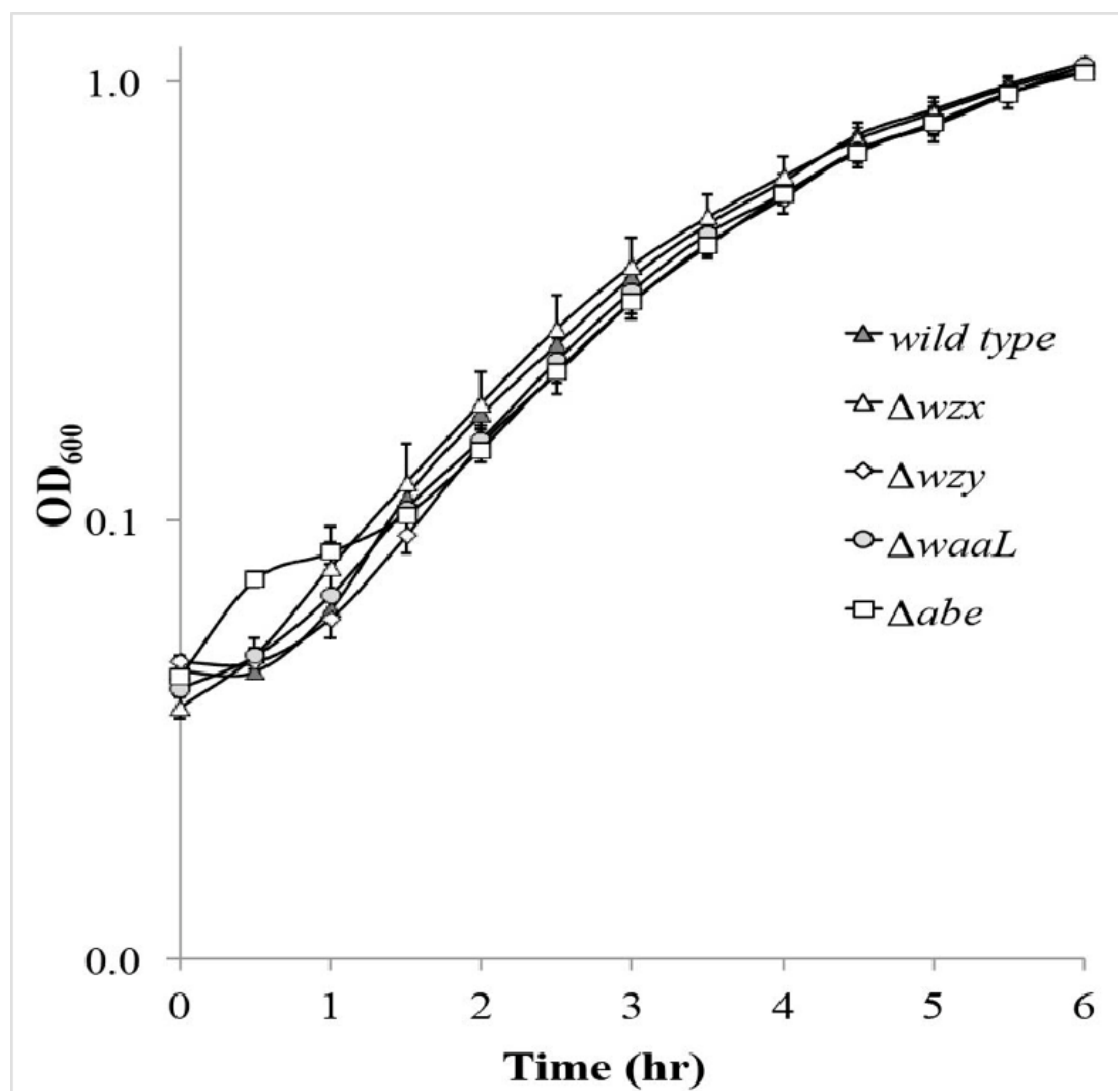

Supplement: Figure S3 [file sph001160055sf3.pdf]
