# Supplementary material for: Model for the Controlled Synthesis of O-Antigen Repeat Units Involving the WaaL Ligase
Source: mSphere. 2015 Dec 30;1(1):e00074-15. doi: 10.1128/mSphere.00074-15 (PMC4863624; doi:10.1128/mSphere.00074-15)
Supplement: Table S1 [file sph001160055st4.pdf]

Strains/ Plasmids	Parent Strain	Genotype/Plasmid Descriptions[1]	Source/Reference
Strains			
P9528	CL4419	*S. enterica* group B serovar Typhimurium strain LT2, *hsdL trp32 nml flaA66, rpsL' xylT404 IlvE452 metE551 metA22 hsdA* Δ*galE*::FRT	(1)
P9529	P9528	CL4419 Δ*galE*::FRT Δ*abe*::*kan*	(1)
P9569	P9528	CL4419 Δ*galE*::FRT Δ*wzx*::*kan*	This study
P9543	P9528	CL4419 Δ*galE*::FRT Δ*wzy*::*kan*	(1)
P9541	P9528	CL4419 Δ*galE*::FRT Δ*waaL*::*cat*	(1)
P9545	P9541	CL4419 Δ*galE*::FRT Δ*waaL*::*cat* Δ*abe*::*kan*	This study
P9574	P9541	CL4419 Δ*galE*::FRT Δ*waaL::cat* Δ*wzx::kan*	This study
P9571	P9529	CL4419 Δ*galE*::FRT Δ*abe*::*kan*/pPR2216	This study
P9568	P9569	CL4419 Δ*galE*::FRT Δ*wzx*::*kan*/pPR2214	This study
P9552	P9543	CL4419 Δ*galE*::FRT Δ*wzy*::*kan*/pPR2198	This study
P9570	P9541	CL4419 Δ*galE*::FRT Δ*waaL*::*cat*/pPR2215	This study
P9575	P9545	CL4419 Δ*galE*::FRT Δ*waaL*::*cat* Δ*abe*::*kan*/pPR2215	This study
P9573	P9574	CL4419 Δ*galE*::FRT Δ*waaL::cat* Δ*wzx::kan*/pPR2214	This study
Plasmids			
pKD3		FRT-flanked *cat* gene, *oriRγ* replicon, ampicillin and chloramphenicol resistance	(2)
pKD4		FRT-flanked *kan* gene, *oriRγ* replicon, ampicillin and kanamycin resistance	(2)
pKD46		lambda recombinase genes (α, β, γ) controlled by arabinose inducible promoter, P_{araB}, temperature sensitive *oriR101* replicon, ampicillin resistance	(2)
pWQ552		Expression vector with p15A *ori*, tetracycline inducible promoter (P_{tet}), ampicillin resistance	(3)
pWQ572		Expression vector with p15A *ori*, tetracycline inducible promoter (P_{tet}), chloramphenicol resistance	(4)
pPR2214		*S. enterica* group B *wzx* gene cloned into pWQ552, ampicillin resistance	This study
pPR2215		*S. enterica* group B *waaL* gene cloned into pWQ552, ampicillin resistance	This study
pPR2216		*S. enterica* group B *abe* gene cloned into pTrc99a, ampicillin resistance	This study
pPR2198		*S. enterica* group B *wzy* gene cloned into pWQ572, chloramphenicol resistance	(5)

[1] Genetic difference from parent strain is underlined, including gene replacements, and plasmid additions.

Reference

1. **Hong Y, Cunneen MM, Reeves PR.** 2012. The Wzx translocases for *Salmonella enterica* O-antigen processing have unexpected serotype specificity. Molecular Microbiology **84:**620-630.
2. **Datsenko KA, Wanner BL.** 2000. One-step inactivation of chromosomal genes in *Escherichia coli* K-12 using PCR products. Proceedings of the National Academy of Sciences of the United States of America **97:**6640-6645.
3. **Willis LM, Stupak J, Richards MR, Lowary TL, Li J, Whitfield C.** 2013. Conserved glycolipid termini in capsular polysaccharides synthesized by ATP-binding cassette transporter-dependent pathways in Gram-negative pathogens. Proceedings of the National Academy of Sciences of the United States of America **110:**7868-7873.
4. **Larue K, Ford RC, Willis LM, Whitfield C.** 2011. Functional and Structural Characterization of Polysaccharide Co-polymerase Proteins Required for Polymer Export in ATP-binding Cassette Transporter-dependent Capsule Biosynthesis Pathways. J Biol Chem **286:**16658-16668.
5. **Hong Y, Morcilla VA, Liu MA, Russell EL, Reeves PR.** 2015. Three Wzy polymerases are specific for particular forms of an internal linkage in otherwise identical O units. Microbiology **161:**1639-1647.
