# Supplementary material for: Model for the Controlled Synthesis of O-Antigen Repeat Units Involving the WaaL Ligase
Source: mSphere. 2015 Dec 30;1(1):e00074-15. doi: 10.1128/mSphere.00074-15 (PMC4863624; doi:10.1128/mSphere.00074-15)
Supplement: Table S2 [file sph001160055st5.pdf]

Primer[1]	Sequence (5'-3')	Detail
7023F	ATG gaattc ATG ACC TTT TTG AAA GAA TAT	Forward primer for cloning *S. enterica* group B1 LT2 *abe* gene, consists of 3b dummy sequence, the *EcoR*I site, and 21b homology to the first 21b of *abe*
7024R	TGA ctgcag TCA TAA CCG TTT CAG TAG TTC	Reverse primer for cloning *S. enterica* group B1 LT2 *abe* gene, consists of 3b dummy sequence, the *Pst*I site, and 21b homology to the last 21b of *abe*
6995F	TGATG gaattc ATG CTA ACC ACA TCA TTA ACG	Forward primer for cloning *S. enterica* group B1 LT2 *waaL* gene, consists of 4b dummy sequence, the *EcoR*I site, and 21b homology to the first 21b of *waaL*
6996R	TACTG ctgcag TTA TCT ATT TCT TAG CGC CAG	Reverse primer for cloning *S. enterica* group B1 LT2 *waaL* gene, consists of 4b dummy sequence, the *Pst*I site, and 21b homology to the last 21b of *waaL*
7021F	AGAC gaattc ATG AAA GTT CAA TTG TTA AAA	Forward primer for cloning *S. enterica* group B1 LT2 *wzx* gene, consists of 4b dummy sequence, the *EcoR*I site, and 21b homology to the first 21b of *wzx*
7022R	CTGT ctgcag TTA TCC CTT ATT TGC CTT AA	Reverse primer for cloning *S. enterica* group B1 LT2 *wzx* gene, consists of 4b dummy sequence, the *EcoR*I site, and 20b homology to the last 20b of *wzx*

[1]Orientations of primers are indicated as: F, forward; and R, reverse.
