# Supplementary material for: Model for the Controlled Synthesis of O-Antigen Repeat Units Involving the WaaL Ligase
Source: mSphere. 2015 Dec 30;1(1):e00074-15. doi: 10.1128/mSphere.00074-15 (PMC4863624; doi:10.1128/mSphere.00074-15)
Supplement: Table S3 [file sph001160055st6.pdf]

Primer [1,2]	Sequence (5'-3') [3]	PCR cassette product and gene target for replacement
6244F	ATGCTCTGGTTATTCCATACCATAGGCTTAACGGAGCGAATTTAGGGATAACAGGGTAAT**GTGTAGGCTGGAGCTGCTTC**	*kan* cassette from pKD4, to replace *galE*
6245R	CGGATGATCGATGGGATTAAATGGGGTCATAACGTCCAAATG**GGAATTAGCCATGGTCC**	
6477F	GCGAATTTAGATGCCACAAGCGTATTTGGAAAGATTCATTAA**ATGGGAATTAGCCATGGTCC**	*cat* cassette from pKD3 to replace *waaL*
6478R	TGGAAAACGCGCTGATACCGTAATAAGTATCAGCGCGTTTTT**GTGTAGGCTGGAGCTGCTTC**	
6248F	GGTTTGAATTTCTAATTCAATTTATTCTATCTGGTGATTGCGTAGGGATAACAGGGTAAT**GTGTAGGCTGGAGCTGCTTC**	*kan* cassette from pKD4, to replace *abe*
6249R	CTAATTTGTTAACGATTTATTTATTAAAAGATCATGAAAATC**TCAGAAGAACTCGTCAAGAA**	
6479F	GATTATAAAAAAGGAATTGAAGAACTACTGAAACGGTTATGA**GTGTAGGCTGGAGCTGCTTC**	*kan* cassette from pKD4, to replace *wzx*
6480R	TTT CTA TTA TAA GTT GGA ATA CAA AAT GAT ATA AGC ATA TGA **CAT ATG AAT ATC CTC CTT AG**	
6481F	GGTAATATTTTTAATACTAAGCATTTTTTCTAAAGGCTCTAT**GTGTAGGCTGGAGCTGCTTC**	*kan* cassette from pKD4, to replace *wzy*
6482R	GCT TCA GAG CCA AAT AAA ACG GCG GCA TTG CCG CCG TAT AAC **TCA GAA GAA CTC GTC AAG AA**	

[1] Orientations of primers are indicated as: F, forward; and R, reverse.
[2] Note that this set of primers was used for strain construction in (1).
[3] Primer sequences: **bold**, priming site for gene cassette; underline, inbuilt I-Sce1restriction site for cassette loss after gene replacement and is only used for optional markerless deletion (2); remainder of sequence corresponds to homology sites used in RED recombination.

Reference

1. **Hong Y, Cunneen MM, Reeves PR.** 2012. The Wzx translocases for *Salmonella enterica* O-antigen processing have unexpected serotype specificity. Molecular Microbiology **84:**620-630.
2. **Stevenson G, Diekelmann M, Reeves PR.** 2008. Determination of glycosyltransferase specificities for the *Escherichia coli* O111 O antigen by a generic approach. Appl. Environ. Microbiol. **74:**1294-1298.
